# Complete Coding Sequence of a Swine Influenza A Variant (H3N2) Virus Isolated in the Republic of Korea in 2017

**DOI:** 10.1128/MRA.01355-19

**Published:** 2020-02-13

**Authors:** Ji Yeong Noh, Van Thi Lo, Young Ji Kim, Sun-Woo Yoon, Dae Gwin Jeong, Woonsung Na, Daesub Song, Hye Kwon Kim

**Affiliations:** a Department of Microbiology, College of Natural Sciences, Chungbuk National University, Cheongju, Republic of Korea; b Infectious Diseases Research Center, Korea Research Institute of Bioscience and Biotechnology, Daejeon, Republic of Korea; c Bio-Analytical Science Division, University of Science and Technology (UST), Daejeon, Republic of Korea; d College of Veterinary Medicine, Chonnam National University, Gwangju, Republic of Korea; e Department of Pharmacy, College of Pharmacy, Korea University, Sejong, Republic of Korea; KU Leuven

## Abstract

Cases of human infection with a swine influenza A virus variant have been reported in the United States, and since 2011, H3N2 variant viruses have also been regularly isolated from swine in the Republic of Korea. Here, we genetically characterized an influenza A H3N2 isolate (A/swine/P17-4/2017). BLASTN analysis of the 8 gene sequences revealed a high degree of nucleotide similarity (97.0 to 99.0%) to porcine strains circulating in the Republic of Korea and the United States. Specifically, we found a high degree of similarity in the nucleotide matrix gene to those of recent isolates from North Carolina. Therefore, continuous epidemiological surveillance is necessary to monitor the variation and evolution of influenza A viruses.

## Abstract (precis)

Cases of human infection with a swine influenza A virus variant have been reported in the United States, and since 2011, H3N2 variant viruses have also been regularly isolated from swine in the Republic of Korea. Here, we genetically characterized an influenza A H3N2 isolate (A/swine/P17-4/2017). BLASTN analysis of the 8 gene sequences revealed a high degree of nucleotide similarity (97.0 to 99.0%) to porcine strains circulating in the Republic of Korea and the United States. Specifically, we found a high degree of similarity in the nucleotide matrix gene to those of recent isolates from North Carolina.

## ANNOUNCEMENT

Influenza viruses are enveloped viruses belonging to the family Orthomyxoviridae that have an 8-segmented, negative-sense, single-stranded RNA genome. H3N2 variant (H3N2v) swine influenza A viruses, which were generated by genetic reassortment between pandemic H1N1 and triple-reassortant H3N2 viruses, have circulated in swine in North America and the Republic of Korea since 1998. The first known human infections with the H3N2v virus containing the pandemic H1N1 matrix (M) gene were reported in the United States in 2011 (1). In this study, we isolated a swine influenza virus and performed genetic characterization of the isolate.

Briefly, six nasal swab samples were collected from grow-finish swine with symptoms of respiratory disease from a swine farm located in Hong Sung, Republic of Korea. Viral RNA from raw samples was extracted with TRIzol LS reagent (Thermo Fisher Scientific, Waltham, MA), and reverse transcription-PCR (RT-PCR) was performed using a universal primer for the influenza M gene (2). One of six samples was confirmed by sequencing to be influenza virus positive. This positive sample was inoculated in specific-pathogen-free (SPF) embryonated chicken eggs for viral isolation. Swine influenza virus (designated A/swine/Korea/P17-4/2017) was isolated with a titer of 10^8^ 50% egg infective dose (EID_50_)/ml.

Viral RNA from the isolated samples was extracted with TRIzol LS reagent and used for cDNA synthesis with Uni12 primer (5′-AGCAAAAGCAGG-3′) (3) and SuperScript III reverse transcriptase (Invitrogen, Carlsbad, CA). A PCR for each segment was performed with a well-established primer set for the eight genomic segments of influenza A viruses (3). The single amplicons were sequenced with Sanger sequencing using a BigDye terminator v3.1 cycle sequencing kit and an Applied Biosystems 3730XL DNA analyzer (Cosmo Genetech Co. Ltd.). Forward and reverse Sanger reads were paired and merged using the BioEdit program. For longer segments (polymerase basic 2 [PB2], PB1, polymerase acidic [PA], and hemagglutinin [HA] genes), internal primers were designed to generate overlapping amplicons and were also Sanger sequenced and merged together. The sequences were assembled and manually edited using the BioEdit program. Complete coding sequences were obtained for all 8 segments encoding the PB1, PB2, PA, HA, nucleoprotein (NP), neuraminidase (NA), matrix (M), and nonstructural (NS) genes.

Sequence similarity was determined using BLASTN (https://blast.ncbi.nlm.nih.gov/), optimizing for highly similar sequences. BLASTN results for each gene indicated an overall high degree of similarity with swine strains from the Republic of Korea (e.g., A/swine/Korea/A18/2011) and the United States. In particular, the nucleotide M gene showed a high degree of similarity (up to 99.0%) with those from recent isolates from North Carolina (Table 1).

**TABLE 1:** BLASTN analysis of viral genomes

Gene	Influenza A virus strain	Query coverage (%)	Sequence identity (%)	GenBank accession no.
PB2	A/swine/Korea/A18/2011 (H3N2)	99	97.89	JX501996
	A/swine/Ohio/11SW162/2011 (H1N2)	99	97.85	KF135537
	A/swine/Korea/KSB/2012 (H3N2)	99	97.85	JX501995
	A/swine/Ohio/11SW174/2011 (H1N2)	99	97.85	CY131552
	A/swine/Ohio/11SW161/2011 (H3N2)	99	97.85	CY131476
PB1	A/West Virginia/06/2011 (H3N2)	100	98.50	JQ290162
	A/swine/Ohio/11TOSU562/2011 (mixed)	100	98.46	KY327745
	A/swine/Indiana/A00968386/2012 (H3N2)	100	98.42	JX549143
	A/swine/Indiana/A00968376/2012 (H3N2)	100	98.42	JX534988
	A/swine/Indiana/A01049964/2011 (H1H2)	100	98.42	JX045974
PA	A/swine/Iowa/A01049723/2011 (H1N2)	100	98.14	KR018892
	A/swine/Korea/KSB/2012 (H3N2)	100	98.14	JX502013
	A/swine/NY/A01104005/2011 (H3N2)	100	98.14	JN940421
	A/swine/Korea/D79/2011 (H3N2)	100	98.09	JX502015
	A/swine/Korea/A18/2011 (H3N2)	100	98.09	JX502014
HA	A/swine/Korea/A18/2011 (H3N2)	100	97.18	JX501999
	A/swine/Illinois/A00857304b/2012 (H3N2)	100	97.12	JQ738179
	A/swine/Korea/D79/2011 (H3N2)	100	97.06	JX502000
	A/swine/Ohio/A01203186/2012 (H3N2)	100	97.06	JX092561
	A/Utah/10/2012 (H3N2)	100	97.00	JX905419
NP	A/Utah/10/2012 (H3N2)	100	98.22	JX905415
	A/Indiana/10/2011 (H3N2)	100	98.15	KJ942595
	A/swine/Ohio/11SW233/2011 (mixed)	100	98.15	KF203223
	A/swine/Korea/CY02-09/2012 (H3N2)	100	98.15	KC471410
	A/swine/Korea/CY03-19/2012 (H3N2)	100	98.15	KC471490
NA	A/swine/Korea/A18/2011 (H3N2)	100	97.66	JX502005
	A/swine/Korea/D79/2011 (H3N2)	100	97.59	JX502006
	A/swine/Indiana/A01049964/2011 (H1N2)	100	97.52	JN652552
	A/swine/Illinois/A01240908/2012 (H3N2)	100	97.45	JX657837
	A/swine/Minnesota/03002/2010 (H1N2)	100	97.45	KY354173
M	A/swine/Iowa/A01244805/2013 (H3N2)	100	98.68	KF478863
	A/swine/North Carolina/A01732036/2016 (H1N2)	100	98.55	KU925694
	A/swine/North Carolina/A02025840/2015 (H1N2)	100	98.55	KT825122
	A/swine/USA/A01475741/2014 (H1N2)	100	98.55	KM044324
	A/swine/Illinois/A01349041/2013 (H1N2)	100	98.55	KC759336
NS	A/swine/Pennsylvania/A01380412/2013 (H3N2)	100	96.39	KF478854
	A/swine/Ohio/11TOSU559/2011 (H3N2)	100	96.25	KY327762
	A/swine/Ohio/11TOSU562/2011 (mixed)	100	96.25	KY327653
	A/swine/Korea/CY03-16/2012 (H3N2)	100	96.25	KC471469
	A/swine/Nebraska/A01049937/2011 (H3N2)	100	96.25	JX860644

**FIG 1:**
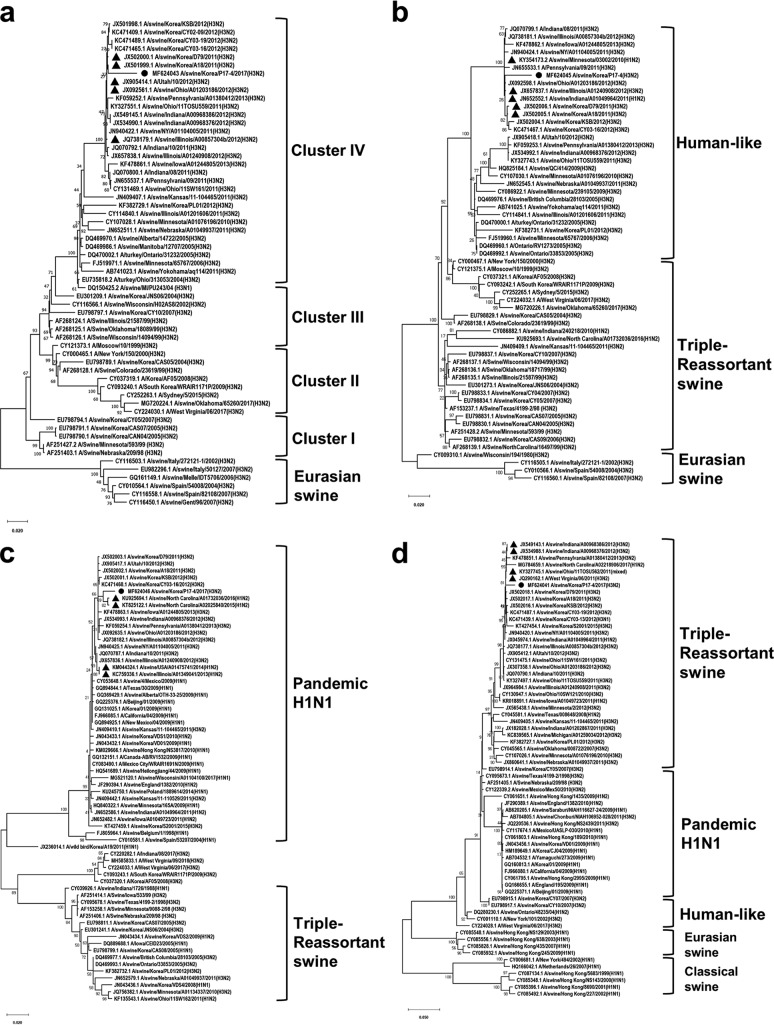
Phylogenetic analysis based on the HA (a), NA (b), M (c), and PB1 (d) gene sequences of the influenza A variant virus A/swine/Korea/P17-4/2017. The phylogenetic tree was generated by the maximum likelihood method with 1,000 replicates of bootstrap sampling and the Kimura 2-parameter model using MEGA 7. The sequence of the isolate in this study is indicated in each panel by a black dot. Sequences showing the highest degree of similarity according to the BLASTN result are denoted by black triangles.

We performed phylogenetic analysis using sequences of influenza virus strains obtained from GenBank that were aligned with BioEdit and then analyzed with MEGA7 using the maximum likelihood method with 1,000 replicates of bootstrap sampling and a Kimura 2-parameter model (4, 5). Our analysis showed that the HA gene of the isolates was closely related to that of isolates from cluster IV of the North American swine virus lineage (triple-reassortant A (H3N2)] (6) (Fig. 1a). Additionally, the NA gene of the isolates belonged to a human-like lineage (Fig. 1b), and the M gene of the isolates was grouped with that of the pandemic H1N1 (pH1N1) 2009 virus (Fig. 1c). Although the PB1 gene of the isolate belonged to a triple-reassortant lineage, the similarity of the PB1 sequence was higher with those of isolates from the United States than with those from the Republic of Korea. According to the Centers for Disease Control, there were reports from 2015 to 2016 of patients infected with influenza A virus variant [A(H3N2)v] (7–9). Furthermore, cases of human infection by influenza variant viruses continue to be reported (10).

Therefore, it is essential to perform recurrent epidemiological surveillance and to continually monitor variations in and evolution of influenza A viruses.

### Data availability.

The genome sequence of the A/swine/Korea/P17-4/2017 variant isolated in this study has been deposited in GenBank under accession numbers MF624040 (polymerase basic 2 [PB2] gene) to MF624047 (nonstructural [NS] gene). Raw sequence reads are available under BioProject accession number PRJNA587049 under SRA accession numbers SRR10394911 to SRR10394918.
